# Corticomotor Plasticity Predicts Clinical Efficacy of Combined Neuromodulation and Cognitive Training in Alzheimer’s Disease

**DOI:** 10.3389/fnagi.2020.00200

**Published:** 2020-07-08

**Authors:** Anna-Katharine Brem, Riccardo Di Iorio, Peter J. Fried, Albino J. Oliveira-Maia, Camillo Marra, Paolo Profice, Davide Quaranta, Lukas Schilberg, Natasha J. Atkinson, Erica E. Seligson, Paolo Maria Rossini, Alvaro Pascual-Leone

**Affiliations:** ^1^Berenson-Allen Center for Noninvasive Brain Stimulation, Division of Cognitive Neurology, Department of Neurology, Beth Israel Deaconess Medical Center and Harvard Medical School, Boston, MA, United States; ^2^Department of Experimental Psychology, University of Oxford, Oxford, United Kingdom; ^3^Department of Geriatrics, Neurosciences and Orthopaedics, Polyclinic A. Gemelli Foundation-IRCCS, Rome, Italy; ^4^Champalimaud Research and Clinical Centre, Champalimaud Centre for the Unknown, Lisbon, Portugal; ^5^Department of Psychiatry and Mental Health, Centro Hospitalar de Lisboa Ocidental, Lisbon, Portugal; ^6^NOVA Medical School – Faculdade de Ciências Médicas de Lisboa, Universidade Nova de Lisboa, Lisbon, Portugal; ^7^Department of Cognitive Neuroscience, Faculty of Psychology and Neuroscience, Maastricht University, Maastricht, Netherlands; ^8^Department of Neuroscience and Neurorehabilitation, IRCCS San Raffaele, Rome, Italy; ^9^Hinda and Arthur Marcus Institute for Aging Research and Center for Memory Health, Hebrew SeniorLife, Boston, MA, United States; ^10^Department of Neurology, Harvard Medical School, Boston, MA, United States; ^11^Guttmann Brain Health Institut, Institut Guttmann, Universitat Autonoma Barcelona, Barcelona, Spain

**Keywords:** clinical trial, randomized controlled, Alzheimer’s disease, transcranial magnetic stimulation, cognitive training, plasticity, combinatory intervention

## Abstract

**Objective:**

To investigate the efficacy of repetitive transcranial magnetic stimulation (rTMS) combined with cognitive training for treatment of cognitive symptoms in patients with Alzheimer’s disease (AD). A secondary objective was to analyze associations between brain plasticity and cognitive effects of treatment.

**Methods:**

In this randomized, sham-controlled, multicenter clinical trial, 34 patients with AD were assigned to three experimental groups receiving 30 daily sessions of combinatory intervention. Participants in the real/real group (*n* = 16) received 10 Hz repetitive transcranial magnetic stimulation (rTMS) delivered separately to each of six cortical regions, interleaved with computerized cognitive training. Participants in the sham rTMS group (*n* = 18) received sham rTMS combined with either real (sham/real group, *n* = 10) or sham (sham/sham group, *n* = 8) cognitive training. Effects of treatment on neuropsychological (primary outcome) and neurophysiological function were compared between the 3 treatment groups. These, as well as imaging measures of brain atrophy, were compared at baseline to 14 healthy controls (HC).

**Results:**

At baseline, patients with AD had worse cognition, cerebral atrophy, and TMS measures of cortico-motor reactivity, excitability, and plasticity than HC. The real/real group showed significant cognitive improvement compared to the sham/sham, but not the real/sham group. TMS-induced plasticity at baseline was predictive of post-intervention changes in cognition, and was modified across treatment, in association with changes of cognition.

**Interpretation:**

Combined rTMS and cognitive training may improve the cognitive status of AD patients, with TMS-induced cortical plasticity at baseline serving as predictor of therapeutic outcome for this intervention, and potential mechanism of action.

**Clinical Trial Registration:**

www.ClinicalTrials.gov, identifier NCT01504958.

## Introduction

Alzheimer’s disease (AD) is the most common neurodegenerative disease (Alzheimer’s Association, 2012) with cognitive decline significantly affecting quality of life (Alzheimer’s Association, 2012). Given that pharmacological agents (Ballard et al., 2011) have limited efficacy and entail unfavorable side effects (Ryu et al., 2005) there is a pressing need for non-pharmacological interventions to complement current options. Importantly, AD is associated with alterations in synaptic function and mechanisms of neuroplasticity (Koch et al., 2012; Rossini et al., 2015), which can be assessed indirectly using transcranial magnetic stimulation (TMS). TMS is a non-invasive technique to generate brief and relatively focal electric currents in the brain via electromagnetic induction. When applied as single or paired pulses to the motor cortex, it provides a diagnostic tool to interrogate cortico-motor reactivity and inhibitory/excitatory intracortical circuits, respectively. As a therapeutic tool, TMS is applied in trains of pulses, termed repetitive TMS (rTMS) to induce changes in excitability of the activated neural circuits that outlast the period of stimulation. TMS can thus be used to modulate activity in brain areas implicated in the cognitive and behavioral dysfunctions of AD. Furthermore, over the last 15 years, a low-intensity form of patterned rTMS, termed intermittent theta-burst stimulation (iTBS), has emerged as a means to induce NMDA receptor (NMDAR)-dependent long-term potentiation (LTP)-like plasticity (Huang et al., 2005, 2007). When applied over the motor cortex, the after-effects of iTBS are measured as the change in the amplitude of motor-evoked potentials (MEPs) elicited by suprathreshold single-pulse TMS, i.e., the change in cortico-motor reactivity, which is thought to reflect cortical plasticity (Pascual-Leone et al., 1994). The after-effects of rTMS and TBS protocols are state-dependent (Silvanto and Pascual-Leone, 2008) and mechanisms underlying rTMS-mediated neuromodulation are similar to neuroplastic effects involved in learning (Cooke and Bliss, 2006). Therefore, targeting neural networks with rTMS while engaging them, for example in cognitive exercises, has been suggested to lead to functional improvements (Freitas et al., 2011) resulting from synergistic effects if repeated in time.

Cognitive training interventions designed to attenuate cognitive decline in dementia typically train basic functions with simple tasks, tapping, for example, into non-verbal and verbal memory, language, and visuo-spatial skills (Hill et al., 2017). Cognitive interventions alone have yielded inconsistent results to date (Clare et al., 2003; Hill et al., 2017; Kallio et al., 2017), while rTMS alone yielded positive results in AD, in a few studies with relatively small samples (Cotelli et al., 2011; Ahmed et al., 2012; Haffen et al., 2012). The concomitant application of these two approaches has shown therapeutic promise in a small industry-sponsored open trial (Bentwich et al., 2011), and three follow-up trials comparing the combined intervention with a control group receiving sham rTMS and sham cognitive training (Rabey et al., 2013; Lee et al., 2015; Rabey and Dobronevsky, 2016). A recent review emphasizes the need for larger studies, and further suggests investigation of treatment-related neurophysiological markers (Buss et al., 2019). Our study builds on and extends these preliminary studies in two important ways. First, we included a control group of AD patients who received sham rTMS with real cognitive training, in order to elucidate effects of cognitive training alone. Furthermore, we aimed to provide mechanistic insight into the intervention by characterizing the neurophysiology of participants using TMS assessments of cortical reactivity, inhibitory/excitatory intracortical circuits, and plasticity, that were assessed longitudinally and also compared at baseline to a group of healthy matched controls (HC). Specifically, we hypothesized that such rTMS-cognitive training-dependent modulation of plasticity is associated with lasting changes in cognition. Moreover, we hypothesized that cortical plasticity is reduced in patients with AD relative to HC, as has been shown in previous studies (Koch et al., 2012; Di Lorenzo et al., 2016) and is modulated by neuronavigated rTMS to multiple brain regions interleaved with region-specific cognitive training.

## Materials and Methods

### Subjects

**FIGURE 1:**
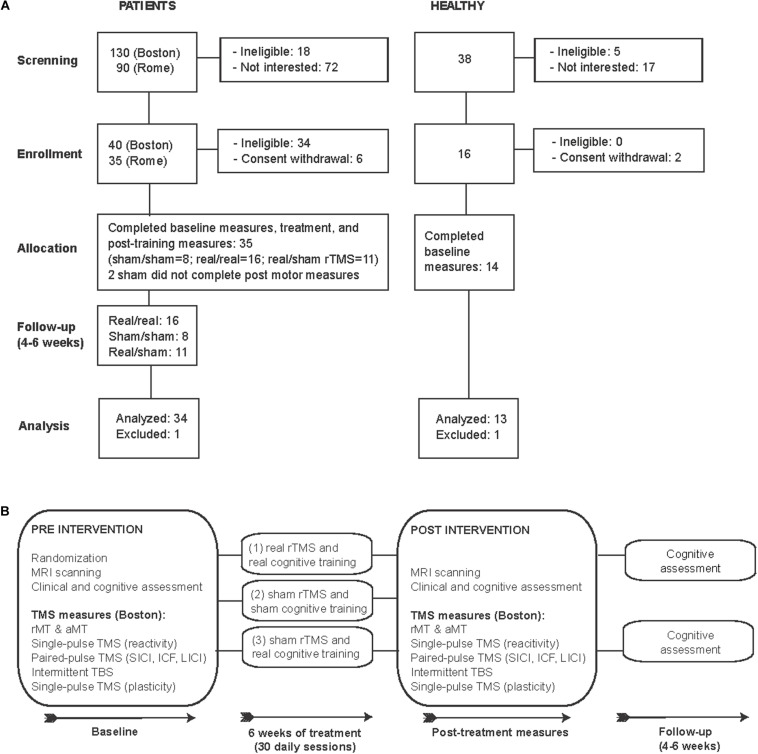
CONSORT flowchart: Enrollment and study design. **(A)** Flow diagram of the enrollment process and final study participants analyzed. **(B)** Schematic representation of study design.

In this proof-of-principle, randomized, double-blind, sham-controlled study we examined 35 patients diagnosed with mild-moderate AD [DSM 5, NIA-AA (McKhann et al., 1984) Figure 1A and Table 1). Patients were randomized using concealed randomization practices (study-independent researcher) and a 2:1:1 group distribution to one of three groups: (1) real cognitive training combined with real rTMS (real/real: *n* = 16), (2) sham cognitive training and sham stimulation over the same brain regions (sham/sham: *n* = 8), or (3) real cognitive training and sham rTMS (real/sham: *n* = 11) (see Figure 1B for an overview of the study design). Except for the technicians applying the intervention, all experimenters and patients were blinded. Findings from two trials, one in Boston (*n* = 21) and one in Rome (*n* = 13), were combined to increase the sample size and reach more reliable conclusions. The eligibility criteria were the following: subjects diagnosed with mild to moderate Alzheimer’s disease according to DSM-5 criteria and criteria established by the NIA-AA (McKhann et al., 1984) for AD, at medium level of certainty according to PET and/or MRI examination; age between 55 and 90 years; written informed consent; MMSE between 18 and 24; normal or corrected ability to see and hear; English (Boston) or Italian (Rome) as primary language. The exclusion criteria were: unstable/chronic medical conditions; major structural/vascular abnormalities, agitation, psychiatric disorders, substance abuse, other progressive neurological disorders different from diseases causing cognitive impairment, or conditions considered a potential hazard for the application of rTMS (Rossi et al., 2009). Patients treated with cholinesterase inhibitors or ginkgo-biloba were allowed to participate if the treatment had started at least 3 months prior to screening and remain stable for the duration of the study. One participant from the real/sham group was excluded due to the incidental finding of a brain lesion. AD patients had a score of 1 (*n* = 30) or 2 (*n* = 4 participants studied in Rome) on the Clinical Dementia Rating scale (CDR), and a Mini-Mental State Examination (MMSE) score between 18 and 24, and 29 of them were medicated (Table 1). We also studied 14 age-matched healthy controls (HC), who underwent the same baseline evaluation as the AD participants in Boston. One HC was excluded as a statistical outlier in several measures. All HC had normal physical, neurological and cognitive exams (CDR = 0 and MMSE score ≥ 28).

**TABLE 1:** Demographic, neuropsychological, morphometric, and neurophysiologic features of study participants (before intervention).

	AD group (*n* = 21, B) (*n* = 13, R)	Healthy controls (*n* = 13, B)	*p*-value	Real/Real intervention (*n* = 16)	Sham/Sham intervention (*n* = 8)	Real/Sham intervention (*n* = 10)	*p*-value
Age (years)*	68.79 ± 7.17	66.00 ± 7.19	0.317	69.25 ± 6.80	67.50 ± 10.27	69.10 ± 5.24	0.710
Gender*	20 Female, 14 Male	7 Female, 6 Male	0.520	12 Female, 4 Male	3 Female, 5 Male	5 Female, 5 Male	0.169
Education (years)*	14.91 ± 4.72	15.62 ± 2.22	0.745	14.25 ± 4.64	17.50 ± 4.00	13.90 ± 5.07	0.064
Medication*				3 MEM 7 COM, 6 AChEI 0 None	0 MEM 2 COM 5 AChEI 1 None	2 MEM 3 COM 1 AChEI 4 None	0.053
SBD left IPL (mm)	24.60 ± 9.78	17.15 ± 2.87	**0.008**	23.50 ± 6.66	26.13 ± 13.49	24.98 ± 12.02	0.961
SBD mean (mm)	18.96 ± 3.57	16.27 ± 2.29	**0.005**	18.36 ± 3.01	20.38 ± 4.17	18.46 ± 4.17	0.555
MMSE*	21.71 ± 2.47	29.46 ± 0.88	**<0.000**	21.19 ± 2.69	20.88 ± 2.95	22.00 ± 1.83	0.662
ADAS-Cog*	23.76 ± 9.90	4.25 ± 1.97	**<0.001**	23.00 ± 9.92	23.61 ± 11.29	25.10 ± 9.67	0.670
ADCS-ADL*	56.77 ± 19.69	74.31 ± 3.45	**0.002**	60.93 ± 15.96	54.67 ± 17.99	51.80 ± 25.64	0.473
GDS	2.43 ± 2.34	0.69 ± 1.11	**0.020**	2.00 ± 2.00	2.67 ± 3.08	3.00 ± 2.35	0.576
rMT	42.33 ± 10.57	46.82 ± 12.33	0.326	44.12 ± 7.02	42.44 ± 14.64	38.60 ± 12.40	0.381
aMT	42.54 ± 9.99	46.42 ± 8.74	0.309	45.53 ± 5.70	43.00 ± 12.74	36.00 ± 12.10	0.179
SICI	0.66 ± 0.39	0.34 ± 0.23	**0.025**	0.63 ± 0.52	0.69 ± 0.20	0.70 ± 0.29	0.784
ICF	1.43 ± 0.85	1.43 ± 0.56	0.611	1.23 ± 0.73	2.10 ± 0.97	0.94 ± 0.26	0.081
LICI	0.59 ± 0.79	0.04 ± 0.06	**0.004**	0.41 ± 0.58	1.06 ± 1.13	0.34 ± 0.43	0.289
MC reactivity	1246 ± 1010	1184 ± 620	0.727	1222 ± 1182	814 ± 473	1815 ± 995	0.203
MC max. plasticity	1.50 ± 0.92	1.87 ± 0.65	**0.020**	1.53 ± 1.34	1.33 ± 0.15	1.66 ± 0.34	0.147
MC mean plasticity	1.09 ± 0.49	1.45 ± 0.60	**0.040**	1.04 ± 0.63	0.99 ± 0.12	1.32 ± 0.44	0.412

*These parameters include the Rome sample (*n* = 34). AChEI, acetylcholinesterase inhibitors; ADAS-Cog, Alzheimer’s Disease Assessment Scale-Cognitive Subscale; ADCS-ADL, Alzheimer’s Disease Cooperative Study–Activities of Daily Living Inventory; B, Boston sample (*n* = 21); COM, combination therapy with AChEI and memantine; GDS, Geriatric Depression Scale; ICF, intracortical facilitation; IPL, inferior parietal lobule; LICI, long-interval intracortical inhibition; MC, motor cortex; MEM, memantine; MMSE, Mini-Mental State Examination; R, Rome sample (*n* = 13); rMT/aMT, resting/active motor threshold; SBD, Scalp-brain distance; SICI, short-interval intracortical inhibition; T5/T10, Time-points at 5 and 10 min post intermittent Theta Burst Stimulation. Group values are presented as mean ± standard deviation (SD), *p*-values are two-tailed. Bold font indicates significant p-values.

Neurophysiological assessment of cortical function was completed in the participants studied in Boston (real/real: *n* = 10; combined sham groups: *n* = 11). Two participants in the sham group did not complete post-intervention measures (due to their distance from the testing site). Interventions took place at the Berenson-Allen Center for Noninvasive Brain Stimulation and the Harvard-Thorndike Clinical Research Center, Beth Israel Deaconess Medical Center, Boston, United States or at the Polyclinic A. Gemelli, Rome, Italy. The local Institutional Review Boards at both institutions (Beth Israel Deaconess Medical Center and Polyclinic A. Gemelli Foundation-IRCCS) approved the study. Participants and their legally authorized representatives (if appropriate) gave written informed consent prior to study onset. AD data from the Boston study originate from the trial registered at ClinicalTrials.gov NCT01504958.

### Cognitive and Behavioral Measures

Cognitive and behavioral functions were assessed within 2 weeks before and 1 week after the therapeutic intervention using tests and inventories drawn from the National Alzheimer’s Coordinating Center – Uniform Data Set (v1) (Beekly et al., 2007). The cognitive subscale of the Alzheimer’s Disease Assessment Scale (ADAS-Cog) was defined as the primary outcome and re-assessed at 1-month follow-up in the Boston and 6 weeks in the Rome sample. The Geriatric Depression Scale (GDS) and the CDR were evaluated at baseline, and the Clinical Global Impression of Change (ADCS-CGIC) was administered after the intervention. To minimize practice/learning effects, alternate forms were used and counterbalanced whenever possible. The ADAS-Cog and ADCS-CGIC and CDR were administered by board-certified neuropsychologists/neurologists, while all other measures, including the Activities of Daily Living (ADCS-ADL) inventory were administered by trained psychometrists. The same experimenter administered the neuropsychological tests at all time points. Participants studied in Rome did not undergo assessment with ADCS-CGIC or GDS.

### Motor Thresholds, Brain Reactivity, and Plasticity

Resting and active motor thresholds (rMT, aMT), motor cortical reactivity, inhibitory/excitatory intracortical circuits, and plasticity were evaluated with TMS combined with electromyography (EMG), at baseline and after the therapeutic intervention (Figure 1B). The eXimia Navigated Brain Stimulation (NBS) system 4 (Nexstim, Finland) was used for single- and paired-pulse stimulation using a handheld figure-of-eight focal biphasic and monophasic coil, respectively. The coils were held over the left motor cortex to elicit motor responses in the contralateral first dorsal interosseous (FDI) muscle. Intensities during assessment of the rMT and aMT were expressed as a percentage of the maximum output of the stimulator. The stimulation was performed positioning the virtual cathode of the coil centered over the site of the scalp to be stimulated and the holder oriented at a 45° angle with respect to the approximate direction of the central sulcus (current flow in postero-anterior direction). The rMT was defined as the minimum intensity of the magnetic field able to generate a motor evoked potential (MEP) of 50 μV in approximately 50% of 10 consecutive stimuli, i.e., generating 5 MEPs out of 10 trials (Rossini et al., 1994, 2015). The aMT was defined as the minimum stimulus intensity that produced a liminal MEP (about 200 μV in 50% of 10 trials) during isometric contraction of the tested muscle.

After assessing rMT and aMT, MEP amplitudes were measured from the right FDI muscle using 30 × 22 mm gel surface electrodes (Ambu, United Kingdom; active over muscle belly, reference over proximal interphalangeal joint of the index finger, ground on the ulnar styloid process). Stimulation intensity for all single-pulse TMS trials was set at 120% of rMT. For paired-pulse TMS, test pulse intensity was set at 120% rMT, while the conditioning pulse intensity and inter-pulse interval (IPI) determined the type of measurement: 80% rMT with a 3-ms IPI to assess short-interval intracortical inhibition (SICI), 80% rMT with a 12-ms IPI to evaluate intracortical facilitation (ICF), and 120% rMT with an IPI of 100 ms to measure long-interval intracortical inhibition (LICI). Intermittent TBS (iTBS) was applied at 80% of aMT. Paired pulse measures were assessed in separate sets (50 pulse pairs with a jittered inter-trial interval of 5–6 s) and expressed as a ratio of the test-MEP to a block of 50 unconditioned pulses. Baseline cortical reactivity (three sets of 30 single TMS pulses) was followed by a 600-pulse regimen of iTBS (20 two-second trains of 50 Hz burst-triplets repeated every 200 ms; MagPro X100, MagVenture A/S, Denmark). Cortical reactivity was reassessed at 5, 10, 20, and 30 min post-iTBS in sets of 30 single TMS pulses. Cortico-motor plasticity was expressed as the percent change from baseline in cortical reactivity. Maximum brain plasticity was defined as the largest of these measures taken at any of the time-points assessed.

### Intervention: Cognitive Training and rTMS

Intervention consisted of daily (monday-to-friday) 1-h sessions of combined cognitive training (real or sham) synchronized with rTMS (real or sham) for 6 weeks (total of 30 sessions) using NeuroAD (Neuronix Medical, Yoqneam, Israel). The NeuroAD system combines brief trains of 10 Hz rTMS targeting brain regions known to be affected in AD (i.e., frontal, temporal, and parietal regions) (Mattson, 2004) interleaved with an adaptive computerized cognitive training program designed to engage the regions targeted by rTMS (Table 2A). Trains of rTMS (20 trains of 2 s, 10 Hz per region per day at 120% rMT) were applied using a handheld figure-of-eight focal coil (SuperRapid stimulator, Magstim Co. Ltd., Whitland, United Kingdom) and were each followed by a cognitive task presented on a touch-screen monitor (20–40 s). During each session, focal stimulation was applied to 3 of 6 predefined brain regions in a pseudo-randomized sequence, without counterbalancing the stimulation sequence between patients. This strategy was devised to ensure the same amount of stimulation per target-region, with each region stimulated 15 times over the course of the intervention. Though rTMS was performed using a focal coil to only one region at a time, the combined effects of stimulating multiple regions over the course of each session could lead to similar effects to those of multifocal stimulation (i.e., concurrent stimulation to several brain regions). The frameless stereotaxic neuronavigation system Brainsight (Rogue Research, Inc., Canada) was used to individually select the targets, guide coil placement, and measure scalp-brain distance, as a surrogate measure of cortical atrophy. The following predefined cortical regions were individually targeted by transforming published group average coordinates into individual MRI space: right/left DLPFC (R/L DLPFC) (Rusjan et al., 2010), right/left IPL (R/L IPL) (Sajonz et al., 2010), Wernicke area in the left superior temporal gyrus (L STG) (Price et al., 2003) and Broca’s area in the left inferior frontal gyrus (L IFG) (Rogalsky et al., 2008). Sham rTMS was delivered with a sham TMS coil (Magstim) that imitated auditory and somatosensory sensations. Regarding cognitive training, for each task, difficulty levels were adapted for each patient, according to performance (Table 2B). The sham cognitive training consisted of a picture preference task using the same stimuli as in the real cognitive training. Participants had to indicate for each picture whether they find it “nice” or “not nice.” The presentation of cognitive stimuli and rTMS were synchronized via the NeuroAD system.

**TABLE 2A:** Cognitive training and morphometric and technical parameters of interest.

Brain Region	L IFG	L STG	R DLPFC	L DLPFC	R IPL	L IPL
**Cognitive tasks**	Sentence similarities, differentiate right/wrong sentences	Differentiate words/pseudo words, assign pictures to categories	Action naming, word recall	Remember color/location of rectangles, word recall	Identify red/blue rectangles	Identify letters B/T/M in a cluster of letters
**Task examples**	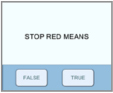	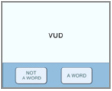	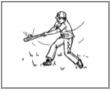	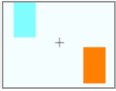	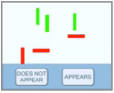	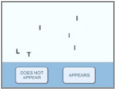

***(A)** Cognitive training tasks were designed to train the following functions: syntax, grammar, lexical meaning, categorization, action naming, object naming, spatial memory, and spatial attention. Cognitive tasks were associated with the stimulation of specific brain areas: L IFG (Broca’s area): determine whether sentences are grammatically correct; L STG (Wernicke’s area): differentiate between words and pseudo words, or to assign pictures to one of two categories; R DLPFC: match written descriptions with pictures; L DLPFC: remember locations and colors of rectangles; R IPL: identify red or blue vertical rectangles within an array of stimuli; L IPL: identify a particular letter in a cluster of random letters. Notably, stimulating several brain areas per session might in turn activate remotely associated hubs of the same networks, possibly leading to a more extensive modulation. For each cognitive task, the difficulty level was adjusted individually and increased following successful completion of a previous level (≥80% correct answers). An increase in difficulty level was either achieved by increasing stimulus-response time, or by increasing the number of displayed target and distracter stimuli. **(B)** Difficulty levels (12 per task) achieved by the real/real and the real/sham group, scalp-brain distances, and stimulation intensity applied per TMS-targeted region. Values are presented as mean standard deviation (SD). **(C)** Correlations between maximum difficulty level achieved during cognitive training tasks per TMS-targeted region with stimulation intensity (real/real group) and with scalp-to-brain distance (real/real and real/sham group). Neurophysiological measures include the Boston sample only (*n* = 21). L IFG, left inferior frontal gyrus; L STG, left superior temporal gyrus; R and L DLPFC, right and left dorsolateral prefrontal cortex; R and L IPL, right and left inferior parietal lobule; SDB, scalp-to-brain distance; t, Kendall’s tau. P-values are one-tailed.*

**TABLE 2B:** Cognitive training and morphometric and technical parameters of interest.

Brain Region	L IFG	L STG	R DLPFC	L DLPFC	R IPL	L IPL	Average
Difficulty level real/real	6.90 ± 2.74	7.40 ± 2.16	6.70 ± 3.06	5.35 ± 3.38	7.90 ± 0.99	6.10 ± 2.81	6.73 ± 1.90
Difficulty level real/sham	5.25 ± 1.56	5.30 ± 2.49	6.40 ± 3.21	5.00 ± 3.45	7.00 ± 3.46	6.20 ± 3.21	5.86 ± 2.62
Scalp-to-brain distance	18.21 ± 5.64	13.29 ± 2.18	21.04 ± 9.50	14.43 ± 3.28	21.78 ± 6.82	23.50 ± 6.66	18.36 ± 3.01
Stimulation intensity (training)	63.92 ± 12.97	63.92 ± 12.97	64.73 ± 14.46	63.92 ± 12.97	64.73 ± 14.46	63.92 ± 12.97	64.19 ± 13.42

*Values are presented as mean ± standard deviation (SD).*

**TABLE 2C:** Cognitive training and morphometric and technical parameters of interest.

Brain Region	L IFG	L STG	R DLPFC	L DLPFC	R IPL	L IPL	Average
τ (difficulty level, stimulation intensity) real/real	0.25	–0.75	0.454	**0.66**	0.48	0.57	0.54
*p*-value	0.486	0.836	0.188	**0.040**	0.161	0.085	0.111
τ (difficulty level, SBD) real/real and real/sham	0.13	–0.46	–0.06	–0.18	**−0.53**	**−0.71**	–0.40
*p* value	0.658	0.087	0.828	0.515	**0.040**	**0.003**	0.136

*L IFG, left inferior frontal gyrus; L STG, left superior temporal gyrus; R and L DLPFC, right and left dorsolateral prefrontal cortex; R and L IPL, right and left inferior parietal lobule; SDB, scalp-to-brain distance; τ, Kendall’s tau. *P*-values are one-tailed. Bold font indicates significant p-values and correlation coefficients.*

### Scalp-Brain Distance Measurements

Scalp-brain distance was defined as the distance between the outer edge of the cortical surface and the outer edge of the scalp and was measured for each target in the plane perpendicular to the scalp tangent (i.e., orthogonal to the plane of the TMS coil) on each individual’s brain MRI for the six stimulated regions and the left motor cortex (site of TMS measures) using Brainsight (Rogue Research Inc., Canada). These measures served as a surrogate indicator of regional brain atrophy (Rusjan et al., 2010) (Table 2B).

### Data Analysis

Data were analyzed using SPSS Statistics 21.0 (SPSS Inc., Chicago, IL, United States) and SAS (version 9.3, SAS Institute, Cary, NC, United States). Significance was set using a 95% confidence interval (α = 0.05).

Individual MEP amplitudes that were greater than 1.5 standard deviations from the mean were excluded. Baseline motor cortical reactivity was defined as the mean MEP amplitude across all 90 trials. To assess plastic changes after iTBS, MEPs from each post-iTBS time point were averaged and divided by baseline. Additionally, the achieved degree of difficulty during cognitive training was assessed for its relationship with atrophy and stimulation intensity.

Group and *post hoc* analyses were calculated with non-parametric Kruskal–Wallis and Mann–Whitney *U* tests, respectively. Correlations [bootstrapped with 10,000 resamples with bias-corrected confidence intervals] were calculated using Pearson’s r tests for whole group analysis and Kendall’s tau tests for subgroup analyses. After finding no difference between the real/sham and sham/sham groups (see section “Results”) both were combined into a single sham rTMS group to increase power. Change in ADAS-Cog (ratio post/pre) was defined as primary outcome and the effect of depression on ADAS-Cog outcome was assessed with a mediation analysis. The difference in ADAS-Cog in the real/real group was tested with a two-sided paired *t*-test (pre vs. post-treatment and pre-treatment vs. follow-up).

Multivariable linear models were built to test either associations between ADAS-Cog and other clinical, demographic, and physiological variables at baseline, or the prediction of the clinical response by said variables, and/or intervention parameters (real vs. sham rTMS and real vs. sham cognitive stimulation). Longitudinal mixed effects models were built to test associations between ADAS-Cog and physiological variables across repeated assessment moments, when adjusting for baseline clinical and demographic variables. Initial models were built according to prior knowledge, and sequential regression models used to test the relevance of additional variables of interest. Data transformations and polynomial models were used to test the better alternative to fit continuous predictors, and relevant interaction terms, namely interactions with active rTMS intervention, were tested. Model assumptions were tested by analyses of residuals, and influence diagnostics were conducted using Cook’s distance.

## Results

### Baseline Comparisons

See Table 1 for an overview of baseline comparisons between AD and HC and across the AD intervention groups. The AD groups did not differ in any of the baseline measures. AD and HC did not differ in age, education, gender, and TMS-measures of rMT, aMT and motor-cortical reactivity. However, depression scores as assessed with the GDS were significantly higher in AD (*U* = 71.5, *z* = −2.41, *p* = 0.020, *r* = −0.41), as was brain atrophy (scalp-brain distance) for the left IPL (*U* = 63.0, *z* = −2.61, *p* = 0.008, *r* = −0.45) and averaged across seven brain regions (*U* = 58.5, *z* = −2.77, *p* = 0.005, *r* = −0.47) (Tables 1, 2B).

**FIGURE 2:**
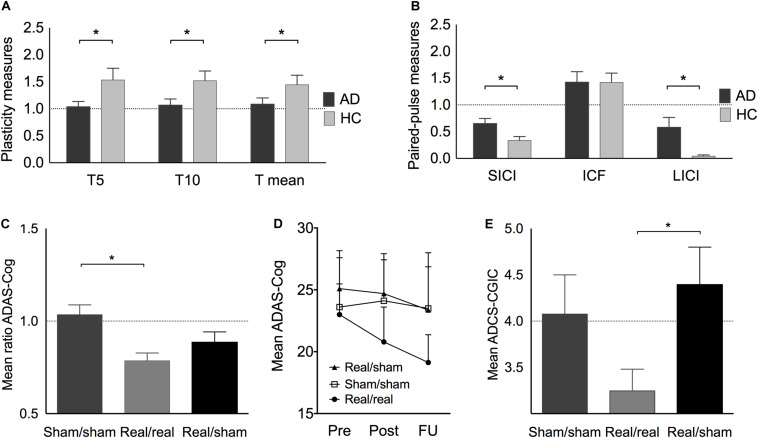
**(A)** Plasticity change at T5, T10, and on average at T5–30 (T mean) expressed as the mean ratios of single-pulse TMS-measured MEP amplitudes pre-post iTBS before the intervention. **(B)** Paired-pulse TMS-measures of short-interval intracortical inhibition (SICI), intracortical facilitation (ICF), and long-interval intracortical inhibition (LICI) in AD (before intervention) and HC. **(C) (including Italian sample):** Best individual score change (ratio post/pre) in ADAS-Cog after real/real (light grey), sham/sham (medium grey), and after combined real cognitive training with sham rTMS (black). **(D)** Average ADAS-Cog score at pre, post and follow-up in the three treatment groups. **(E)** Average ADCS-CGIC scores. A score of 4 (dotted line) is equivalent to no change from pre- to post-intervention, scores < 4 indicate improvement, scores > 4 indicate decline. Indicated values correspond to mean ± standard error (SE).

Plasticity indices measured up to 30 min after iTBS were significantly different between AD patients and HC at T5 (*U* = 183, *z* = 2.13, *p* = 0.033, *r* = 0.37), T10 (*U* = 179, *z* = 1.98, *p* = 0.048, *r* = 0.35), and averaged over T5 to T30 (*U* = 181, *z* = 2.06, *p* = 0.040, *r* = 0.36) (Figure 2A). Furthermore, the maximum plasticity change (*U* = 187.5, *z* = 2.302, *p* = 0.020, *r* = 0.40) was significantly smaller in AD. ICF was similar in AD and HC, while SICI and LICI were significantly reduced in AD (SICI: *U* = 56, *z* = −2.23, *p* = 0.025, *r* = −0.40; LICI: *U* = 42, *z* = −2.81, *p* = 0.005, *r* = −0.50) (Figure 2B).

After adjusting for a 5% False Discovery Rate (FDR), non-significant trends were observed for plasticity indices at T5 and T10, as well as overall and maximum plasticity change (*p* < 0.1), while differences in SICI and LICI remained significant (*p* < 0.05). Furthermore, atrophy of the left IPL and average atrophy remained significantly larger in AD (*p* < 0.05).

### Intervention Effects in Alzheimer Disease Patients

The largest score change in ADAS-Cog within 4–6 weeks after the end of intervention was significantly different between groups (*H*[2] = 10.16, *p* = 0.006) (Figure 2C). *Post hoc* tests revealed a significantly higher improvement in the real/real group as compared to the sham/sham (*U* = 115, *z* = 3.13, *p* = 0.001, *r* = 0.64) but not the real/sham group (*U* = 110, *z* = 1.56, *p* = 0.121, *r* = 0.31). Immediately after the intervention, the real/real group improved their cognitive score on average by 2.18(±3.70) points, while the real/sham group improved by 0.4(±4.14) points and the sham/sham group showed a decline of 0.49(±2.48) points. During the following 4–6 weeks post-intervention, the real/real group continued to improve on average by an additional 1.69(±4.07) points, that is, at the end of the follow-up period the real/real group improved on average by 3.87(±4.54) points from baseline (Figure 2D). Notably, the immediate change from pre to post-treatment in the real/real group reached statistically significant levels (*t*(15) = 2.408, *p* = 0.029, CI 95% [0.253, 4.16]) as well as the change from pre to follow-up (*t*(15) = 3.413, *p* = 0.004 CI 95% [1.45, 6.29]). Neither of the sham groups (real/sham and sham/sham) showed a significant cognitive change from pre to post (Supplementary Table S2). Importantly, two-thirds of the patients in the real group continued to improve in ADAS-Cog even after the end of intervention. Comparing across groups, a marginally significant difference was observed in ADCS-CGIC (real/real: 3.25 ± 0.72, sham/sham: 4.08 ± 1.02, real/sham: 4.40 ± 0.89; *H*[2] = 5.950, *p* = 0.051). We thus decided to explore post-hoc results and found a significantly larger improvement for the real/real as compared to the real/sham group (*U* = 42, *z* = 2.21, *p* = 0.04, *r* = 0.57) (Figure 2E), while the other group comparisons did not reach significance. The score change in ADCS-ADL immediately after the end of the intervention was significantly different between groups (*H*[2] = 6.95, *p* = 0.031). *Post hoc* tests showed that the real/real group (*U* = 18, *z* = −2.12, *p* = 0.036, *r* = −0.46) as well as the real/sham group (*U* = 53, *z* = 2.5, *p* = 0.011, *r* = 0.63) showed a significantly better outcome than the sham/sham group. The real/real and real/sham groups did not differ significantly in ADL outcome (*U* = 89, *z* = 0.79, *p* = 0.461, *r* = 0.16).

As hypothesized, the two sham groups did not differ significantly with regard to their largest ADAS-Cog score change (*U* = 23, *z* = −1.56, *p* = 0.122, *r* = 0.34) and their outcomes in ADCS-CGIC (*U* = 19, *z* = 0.76, *p* = 0.537, *r* = 0.23). We therefore followed our strategy in accordance with the 2:1:1 group randomization and further compared the real/real group (*n* = 16) with the combined sham groups (*n* = 18) in order to assess rTMS-specific intervention effects and increase statistical power. In these analyses the difference for ADAS-Cog score change remained significant (*U* = 225, *z* = 2.78, *p* = 0.004, *r* = 0.48) and the change in ADCS-CGIC (sham *n* = 11, real/real *n* = 10) became significant (*U* = 87, *z* = 2.35, *p* = 0.024, *r* = 0.51). Moreover, the change in ADAS-Cog was correlated with clinical changes as indicated by CGIC (*r* = 0.473, *p* = 0.031, 95% CI 0.08 to 0.75). Furthermore, a mediation analysis with bootstrapping (10,000 samples, Preacher and Hayes, 2008), to examine the effect of depression on intervention outcome, showed that depression scores did not account for the variance in ADAS-Cog (*R*^2^ = 0.15, *F*_(2,18)_ = 1.625, *p* = 0.225; 95% bias corrected confidence interval for the mediating variable: [−0.007 to 0.037]). No major side effects were reported in any participants.

### Association of Cognitive With Physiological and Other Measures

At baseline, ADAS-Cog (lower values indicate better cognitive function) was significantly correlated with rMT (*r* = −0.44, *p* = 0.009, 95% CI −0.64 to −0.20), SICI (*r* = 0.564, *p* = 0.001, 95% CI 0.21 to 0.77) and marginally with LICI (*r* = 0.304, *p* = 0.096, 95% CI 0.03 to 0.71), as well as atrophy in the left IPL (*r* = 0.42, *p* = 0.013, 95% CI 0.09 to 0.70), and mean atrophy (*r* = 0.37, *p* = 0.033, 95% CI 0.00 to 0.68). Atrophy in the motor cortex was positively correlated with rMT (*r* = 0.389, *p* = 0.023) across all participants. Cortical reactivity and plasticity were not associated with cortico-motor atrophy (*p*_*s*_ > 0.05). The maximum difficulty level reached in region-specific tasks was significantly correlated with absolute TMS intensity for the left DLPFC (*r* = 0.66, *p* = 0.04) in the real/real group and with atrophy in the left (*r* = −0.707, *p* = 0.003) and the right IPL (*r* = −0.534, *p* = 0.04) (Table 2C).

**FIGURE 3:**
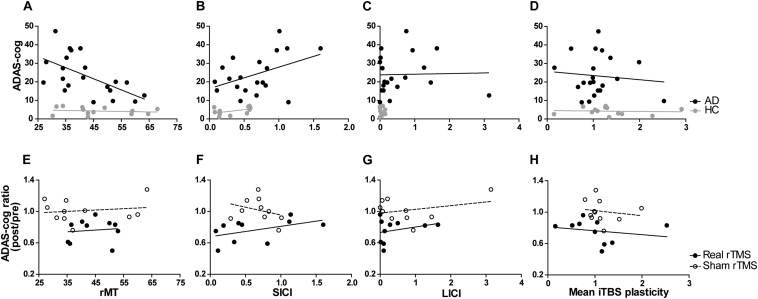
Relationship between TMS-driven measures and cognitive function (ADAS-Cog). Relationship at baseline **(A–D)** in AD and HC, or clinical response over time (ADAS-Cog ratio) in AD patients **(E–H)** treated with real or sham rTMS. Cortico-motor plasticity was expressed as the change of cortical reactivity from baseline to post-iTBS. Circles reflect data for individuals and lines are unadjusted regression lines for the specified groups. When adjusting for age, education and mean atrophy, significant associations were found between baseline ADAS-Cog and resting motor threshold [rMT – **(A)**], short-interval intracortical inhibition [SICI – **(B)**], long-interval intracortical inhibition [LICI – **(C)**] and mean plasticity from 5 to 30 min post-iTBS **(D)**. However, in models adjusting for rTMS intervention (real vs. sham), mean plasticity **(H)**, but not rMT **(E)**, SICI **(F),** and LICI **(G)** was found to be a significant predictor of response.

In multivariable linear regression analyses with data from all participants at baseline, the association of ADAS-Cog with rMT and SICI was confirmed, and found also for aMT, mean plasticity indices averaged over T5–T30, maximum plasticity, and LICI (Table 3 and Figure 3). None of these associations were confounded by mean atrophy. However, the associations between baseline ADAS-Cog and plasticity measures, as well as LICI, were no longer significant when adjusting for diagnosis. In additional linear regression analyses of ADAS-Cog ratio (post-intervention/baseline) as a measure of response to treatment in patients with AD, when adjusting for rTMS intervention, a significant predictor of response in all models, age, education, gender, and cognitive training were not significant predictors of response. However, mean atrophy and atrophy over the left DLPFC, but not other cortical areas, were found to be significant predictors of response (Table 3). Importantly, in similar rTMS adjusted analyses, MEP amplitude, as well as plasticity at T5 and T10, mean plasticity from T5 to T30 and maximum plasticity, were all found to be significant predictors of response (Table 3 and Figure 3). Interaction terms with rTMS were not significant and none of these associations were confounded by cognitive stimulation. However, plasticity at T5 and maximum plasticity became only marginally significant predictors of clinical response when adjusting for mean atrophy. The effects for the remaining predictors were not significantly affected.

**TABLE 3:** Parameter estimates and model fit statistics for multivariable linear models testing associations between TMS-driven measures of cortico-motor reactivity evaluated at baseline, and cognitive function (ADAS-Cog) also measured at baseline, or clinical response (ADAS-Cog ratio) in the sample from Boston (*n* = 21).

Linear Models							

Dependent variable		Baseline ADAS-Cog (AD and HC)			ADAS-Cog ratio (post-intervention/baseline; AD only)		
		
Base model	Independent variables	Beta ± SE	*p*	*R*^2^	Beta ± SE	*p*	*R*^2^
	Age (years)	0.3 ± 0.3	0.3	**0.09**			
	Education (years)	0.3 ± 0.7	0.6				
	Mean SBD	**1.3 ± 0.6**	**0.04**				
	rTMS				**−0.25 ± 0.06**	**<0.001**	**0.42**
Single clinical or demographic variables added to base model	Diagnosis	**−18 ± 3.6**	**<0.0001**	**0.5**			
	Cognitive stimulation				−0.1 ± 0.08	0.1	0.47
	Age (years)				−2.4*10^–4^ ± 4.6*10^–3^	0.96	0.39
	Gender (male)				−0.02 ± 0.07	0.8	0.39
	Education (years)				6.9*10^–3^ ± 8.8*10^–3^	0.4	0.41
	Mean SBD				−0.02 ± 8.7*10^–3a^	**0.02**	**0.54**
Single structural variables (scalp-brain distance) added to base model	Left DLPFC				−0.02 ± 0.01^a^	**0.03**	**0.51**
	Right DLPFC				−5.2*10^–3^ ± 4.3*10^–3^	0.2	0.44
	Left IFG				−3.2*10^–3^ ± 5.5*10^–3^	0.6	0.4
	Left IPL				−3.6*10^–4^ ± 3.4*10^–3^	0.9	0.39
	Right IPL				−2,6*10^–3^ ± 4*10^–3^	0.5	0.41
	MC				−6.1*10^–3^ ± 0.01	0.6	0.4
	Left STG				−4.6*10^–3^ ± 5.3*10^–3^	0.4	0.42
	Mean distance (7 areas)				−0.02 ± 8.7*10^–3a^	**0.02**	**0.54**
Single physiological variables added to base model	rMT	**−0.7 ± 0.2**	**<0.001**	**0.42**	1.8*10^–3^ ± 3.2*10^–3^	0.6	0.4
	aMT	**−0.7 ± 0.2**	**0.001**	**0.35**	2.4*10^–3^ ± 3.4*10^–3^	0.5	0.41
	MEP amplitude	1.9*10^–3^ ± 2.5*10^–3^	0.4	0.08	−1*10^–4^ ± 4*10^–5a^	**0.02**	**0.56**
	Plasticity T5	−4.3 ± 3.9	0.3	0.08	**−0.2 ± 0.1^a,b^**	**<0.05**	**0.5**
	Plasticity T10	−8.7 ± 4.3^a^	0.06	0.25	**−0.2 ± 0.08^a^**	**0.02**	**0.56**
	Mean Plasticity T5-T30	**−8.9 ± 4.3^a^**	**<0.05**	**0.26**	**−0.3 ± 0.1^a^**	**0.02**	**0.54**
	Maximum plasticity T5-T30	**−9.9 ± 4.1^a^**	**0.02**	**0.31**	**−0.2 ± 0.09^a,b^**	**<0.05**	**0.52**
	SICI	**19.5 ± 5.1**	**<0.001**	**0.39**	0.08 ± 0.09	0.4	0.42
	ICF	−1.9 ± 3.2	0.6	0.06	0.02 ± 0.04	0.7	0.39
	LICI	**12.2 ± 4.4^a^**	**0.01**	**0.34**	0.05 ± 0.04	0.2	0.43

*^*a*^One or more subjects were not included in this model due to being influential observations according to Cook’s distance analysis. ^*b*^Becomes only marginally significant (0.05 < *p* < 0.08) when adjusted for mean scalp-brain distance. ADAS-Cog, cognitive subscale of the Alzheimer’s Disease Assessment Scale; ICF, intracortical facilitation; LICI, long-interval intracortical inhibition; MEP, motor evoked potential; rMT/aMT, resting/active motor threshold; rTMS, repetitive transcranial magnetic stimulation; SBD, Scalp-brain distance; SICI, short-interval intracortical inhibition; T5/T10/T30, Time-points at 5 10 and 30 min post intermittent Theta Burst Stimulation. Bold font indicates significant values.*

Even though change in neurophysiological parameters exploring plasticity from pre to post-treatment did not differ significantly between groups (*H*[2] = 0.464, *p* = 0.793) (Supplementary Table S1), longitudinal regression models were built to test physiological and other factors associated to ADAS-Cog across intervention. These multivariable analyses were adjusted for rTMS, that was again a significant parameter in the models. In separate rTMS-adjusted models, age, gender, education, cognitive stimulation and mean atrophy were not associated to ADAS-Cog, and the latter two variables were also not confounders in any of the subsequent models. As found at baseline, rMT and aMT were significantly associated to ADAS-Cog, but the associations with SICI, LICI, and T5 and T10 plasticity measures were no longer significant in these longitudinal analyses. However, the association between ADAS-Cog and both mean and maximum plasticity from T5 to T30 remained significant. Importantly, these were also the only instances where an interaction term between the physiological measure and rTMS was also significant, showing not only that these associations between cognition and physiology persist across intervention, but also that they were modulated by active rTMS treatment (Table 4).

**TABLE 4:** Parameter estimates and model fit statistics for multivariable mixed effects longitudinal models testing associations between TMS-driven measures of corticomotor reactivity and cognitive function (ADAS-Cog), measured across time (pre and post-treatment), in the sample from Boston (*n* = 21).

Longitudinal Mixed Effects Models				

Dependent variable: ADAS-Cog (AD only)				

	Independent variables		Beta ± SE	*p*
Base model	rTMS		−10.5 ± 3.8	0.006
	Time (pre- vs. post treatment)		−2.8 ± 0.9	0.002
Single clinical or demographic variables added to base model	Cognitive stimulation		−2.5 ± 5.4	0.6
	Age (years)		−0.07 ± 0.3	0.8
	Gender (male)		4.8 ± 4	0.2
	Education (years)		0.1 ± 0.5	0.9
	Mean scalp-brain distance		−0.3 ± 0.6	0.5
Single physiological variables added to base model	rMT		−0.5 ± 0.2	0.004
	aMT	aMT	1.9 ± 0.9	<0.05
		aMT^2^	−0.02 ± 0.01	0.03
	MEP amplitude		4.7*10^−4^ ± 6.4*10^−4^	0.5
	Plasticity T5		1.5 ± 1.2	0.2
	Plasticity T10		−0.4 ± 1.3	0.7
	Mean Plasticity T5–T30	MeanT5-T30	5.2 ± 2.2	0.02
		MeanT5-T30*rTMS	−6 ± 2.6	0.02
	Maximum Plasticity T5–T30	MaxT5-T30	4.9 ± 1.7	0.005
		MaxT5-T30*rTMS	−5.2 ± 1.9	0.005
	SICI^a^		3.4 ± 2.8	0.2
	ICF		−0.4 ± 0.8	0.6
	LICI		−0.5 ± 1.5	0.7

*^*a*^One or more subjects were not included in this model due to being influential observations according to Cook’s distance analysis. ADAS-Cog, cognitive subscale of the Alzheimer’s Disease Assessment Scale; ICF, intracortical facilitation; LICI, long-interval intracortical inhibition; MEP, motor evoked potential; rMT/aMT, resting/active motor threshold; rTMS, repetitive transcranial magnetic stimulation; SICI, short-interval intracortical inhibition; T5/T10/T30, Time-points at 5 10 and 30 min post intermittent Theta Burst Stimulation.*

## Discussion

The primary aim of the present study was to assess the efficacy of rTMS combined with cognitive training for the treatment of cognitive symptoms in AD patients. Furthermore, we aimed to analyze if cortical plasticity was different in patients with AD versus HC as shown in previous studies, whether its modulation by the combined rTMS-training intervention was possible, and whether it might be associated with changes in cognitive function. For the primary aim, in order to reach a suitable sample size, findings from two parallel trials utilizing the same recruitment criteria, interventions, and cognitive assessments (Boston and Rome) were combined.

### Baseline Measures

At baseline, we found no significant difference in rMT and aMT between patients with AD and HC. However, both were robust indicators of severity of cognitive dysfunction, even when controlling for brain atrophy. Cortico-motor plasticity revealed significant differences between AD patients and HC, as could be expected from findings of a positive correlation between amyloid-β cerebrospinal fluid levels and long-term potentiation (LTP)-like effects induced by iTBS (Mori et al., 2011). Another study (Koch et al., 2012) showed significant differences in brain plasticity between AD and HC at several time-points between 11 and 25 min after iTBS. We found significant differences for T5, T10, average plasticity (T5–30), and for maximum iTBS-induced plasticity, as well as significant linear associations between all of these plasticity measures and ADAS-Cog at baseline, when adjusting for several demographic and clinical factors, as well as atrophy. Change in neurophysiological parameters exploring plasticity from pre to post-treatment did not differ significantly between AD groups. The differential time-course of post-iTBS plasticity could be related to the fact that our patients were mostly medicated, while Koch et al. (2012) investigated medication-naïve patients. Huang et al. (2007) found that memantine significantly impacts measures of TBS-induced plasticity in normal subjects, which might have played a role for some patients. And in an own study, we found that pharmacological treatment can also have a specific impact on plasticity measures in AD (Brem et al., 2013).

We also found reduced SICI and LICI in AD patients, which were associated to baseline ADAS-Cog. LICI has been shown to interact with short-latency afferent inhibition (SAI) in healthy subjects (Udupa et al., 2009), which again has been shown to be reduced in AD (Koch et al., 2012), and may be a valuable biomarker that could be assessed in future studies using TMS-EEG across regions of the cortical convexity (Farzan et al., 2010). SICI has been previously reported to be reduced in AD patients and to be related to disease severity (Liepert et al., 2001), however, findings are divergent (Freitas et al., 2011). Atrophy over the primary motor cortex was not associated with cortico-motor reactivity and plasticity, and mean atrophy did not confound the associations between cognitive and motor measures. Atrophy in AD was significantly larger, specifically for the left IPL, which is in accordance with previous findings stating that IPL is among the first brain regions to show atrophy (Jacobs et al., 2011).

### Clinical Effect

Overall training progression did not differ between the two groups who received active cognitive training, indicating that the addition of rTMS to cognitive training is crucial to achieve cognitive improvements and not progression in the training. High-frequency rTMS protocols (such as the 10 Hz stimulation protocol used in the treatment phase of the present study) are established as a safe and effective method to increase brain excitability and have been applied in a wide range of patient populations in a similar way as in the present study (Bentwich et al., 2011; Cotelli et al., 2011; Chang et al., 2012; Kakuda et al., 2013; Khedr et al., 2014; Yang et al., 2013; Kim et al., 2014; Wobrock et al., 2015; McGirr et al., 2015; Quan et al., 2015). The degree of improvement in the real/real group is clinically meaningful (Schrag et al., 2012) and was not mediated by potential effects of rTMS on depression. Previous studies using the same approach (Bentwich et al., 2011; Rabey et al., 2013; Lee et al., 2015; Rabey and Dobronevsky, 2016) also reported similar changes in ADAS-Cog, which further strengthens our findings. Interestingly, substantial cognitive improvement occurred after the end of intervention, possibly reflecting time-prolonged modulation of neurophysiological processes underlying cognitive maintenance. After combining the two sham groups, an improvement in the ADCS-CGIC in the real intervention group that was associated with cognitive improvement became significant, stressing the importance of the addition of rTMS. Both the real/real and the real/sham group improved significantly more in ADL than the sham/sham group after the intervention.

In contrast to previous studies (Bentwich et al., 2011; Rabey et al., 2013; Lee et al., 2015; Rabey and Dobronevsky, 2016), we used neuronavigation to ensure precise targeting of the desired cortical brain regions. Furthermore, we demonstrated that sham rTMS combined with real cognitive training (real/sham group) showed similar effects as the sham/sham group, emphasizing the importance of rTMS. Though, we cannot establish whether rTMS in combination with cognitive training produces the same effect as rTMS on its own, we strongly believe that the combination is crucial (Brem and Sensi, 2018). Added efficacy might arise from Hebbian mechanisms of synaptic reinforcement induced by the combined impact of cognitive training and rTMS on the same neural networks engaged in the different tasks. This is supported by the demonstrated cortico-motor reactivity and plasticity effects in the participants studied in Boston. Blinding is difficult to assess given the nature of this cohort. Nonetheless, when asked to indicate group assignment among the participants studied in Boston, 6/10 patients (60%) in the real/real group and 6/11 patients in the sham groups (55%) thought they had received real intervention. However, as no electrical surface electrodes were used in the sham protocol, we cannot entirely exclude non-neural effects.

Our patients progressed less in training tasks related to the left and right IPL the greater the cortical atrophy in these regions. It is important to consider that greater atrophy would have resulted in relatively lower rTMS intensity at cortical level in IPL. Furthermore, training gains were significantly correlated with absolute TMS intensity for the left DLPFC. Therefore, considering that atrophy was a negative predictor of clinical response, future studies may base stimulation intensity on the modeling of current distribution (Wagner et al., 2008). Notably, the activation of a broad range of brain areas known to be associated with AD pathology was thought to maximize cognitive effects. Though we did not find clear benefits of cognitive training alone, we nevertheless observed numerical improvements across a range of parameters. Optimizing and individualizing the cognitive training could possibly improve interaction effects with rTMS and enhance clinical outcomes.

### Correlation of Physiological Measures With Cognitive Performance Across Treatment

Transcranial magnetic stimulation has been previously found to be precise enough to track disease-related neuroplastic changes of motor cortex output in early AD (Ferreri et al., 2003). Notably, in our study, MEP amplitude, as well as plasticity at T5 and T10, mean and maximum plasticity were all significant predictors of clinical response, even when adjusting for mean atrophy and other intervention parameters. Furthermore, the association between ADAS-Cog and several plasticity measures, as well as motor thresholds, was preserved across the intervention resulting in significant cognitive improvement. Importantly, in the case of plasticity measures, active rTMS intervention was a significant modulator of this association. Thus, we found that measures of iTBS-induced plasticity were associated to cognitive function not only at baseline but also after intervention, in an rTMS-dependent fashion.

During the intervention, rTMS was not applied to the motor cortex, and yet this was the cortical area from which plasticity measures were collected. The observed correlations between cortico-motor plasticity and cognitive measures strongly suggest that alterations in plasticity may occur globally. Further support for this assumption is suggested by the positive correlation found between maximum level achieved in training tasks and stimulation intensity. A neuroimaging study has shown that memory training can drive non-motor brain activation patterns of patients with mild cognitive impairment toward normalization (Belleville et al., 2011). Changes in motor cortical measures after stimulation of non-motor areas have been reported previously (Pallanti et al., 2012), as has prediction of non-motor effects of prefrontal stimulation by motor cortical measures (Oliveira-Maia et al., 2017). These effects could arise through changes in overlapping subcortical glutamatergic and dopaminergic pathways (Wang et al., 2012), which in turn could be driven via cortico-subcortical projections from stimulated non-motor areas.

### Study Limitations

This study did not assess the effects of rTMS alone, which would have necessitated adding a fourth study arm. Though magnetic stimulation alone could affect cognition without additionally inducing brain activity (Wang et al., 2019), the combination of rTMS with cognitive interventions is thought to be more effective (Brem and Sensi, 2018). Given our main interest in the combined method, we actively decided against using resources for an additional study arm and to favor the inclusion of two rTMS sham groups (2:1:1 randomization). The challenges in enrolling AD patients in such an intensive protocol (daily treatment for 6 weeks) account for the modest sample size. Though the two study sites used slightly different criteria, we believe that the effects of a center bias on the primary outcome are minimal as results remained largely unchanged after adding the results from Rome to the results from Boston (Supplementary Results). However, the patients in Rome did not undergo neurophysiological measures, which might have had an impact on compliance, tiredness at pre- and posttest and likelihood to adhere to the study plan. Finally, although we chose a control cognitive task with minimal cognitive engagement in order to avoid having the control condition represent an “uncontrolled brain state,” it is possible that changes in brain state could have lead to unspecific improvements in the sense of an active control training.

## Conclusion

We found that the combinatory treatment of rTMS and cognitive training resulted in significant cognitive improvement as assessed with the ADAS-Cog, while cognitive training alone did not lead to significant improvement compared to placebo treatment. Confirming prior reports, we show abnormalities in the mechanisms of plasticity and cortical reactivity in patients with AD, and show for the first time that these can be modulated by a combined non-invasive stimulation and cognitive training paradigm resulting in behavioral, clinical and cognitive benefits. This study supports the relevance of non-pharmacological combinatory interventions in individuals with AD and provides important groundwork for future studies to build upon. Many questions remain to be answered, including how cognitive improvements translate into patients’ daily lives and whether neurophysiological measures could act as potential biomarkers for interventions in AD. Future research should assess a broader range of patients and aim to improve applicability in the clinical environment.

## Data Availability Statement

The datasets generated for this study are available on request to the corresponding author.

## Ethics Statement

The studies involving human participants were reviewed and approved by the local Institutional Review Boards at both Beth Israel Deaconess Medical Center and Polyclinic A. Gemelli Foundation-IRCCS. The patients/participants provided their written informed consent to participate in this study.

## Author Contributions

A-KB and AP-L designed the study in Boston. CM, PP, and PR designed the study in Rome. A-KB, LS, NA, and ES collected the data in Boston. RD and DQ collected the data in Rome. A-KB, PF, AO-M, LS, NA, and ES analyzed the Boston data. RD, CM, PP, and DQ analyzed the Rome data. A-KB prepared the initial draft report. AP-L edited the report, which was critically reviewed by all authors. A-KB, PR, and AP-L had full access to all the data in the study and take responsibility for the integrity of the data and the accuracy of the data analysis.

## Conflict of Interest

AO-M is recipient of a grant from Schuhfried GmBH for norming and validation of cognitive tests, and national coordinator for Portugal of a non-interventional study (EDMS-ERI-143085581, 4.0) to characterize a treatment-resistant depression cohort in Europe, sponsored by Janssen-Cilag Ltd, and trial of a psilocybin therapy for treatment-resistant depression, sponsored by Compass Pathways, Ltd. (EudraCT NUMBER: 2017-003288-36). AP-L serves on the scientific advisory boards for Starlab Neuroscience, Neuroelectrics, Constant Therapy, Cognito, and Neosync; and is listed as an inventor on several issued and pending patents on the real-time integration of transcranial magnetic stimulation (TMS) with electroencephalography (EEG) and magnetic resonance imaging (MRI). The remaining authors declare that the research was conducted in the absence of any commercial or financial relationships that could be construed as a potential conflict of interest.

## Supplementary Material

TABLE S1 Plasticity measures in the AD sample from Boston (*n* = 21) at baseline and after the end of the intervention (T5, T10, T20, T30).

TABLE S2 ADAS-Cog results from each site (Boston and Rome) and combined results.

RESULTS Before adding the results from Rome to the results from Boston, we find very similar results. In Boston, the largest score change in ADAS-Cog within one month after the end of intervention was significantly different between groups (Boston: *H*[2] = 11.626, *p* = 0.003; Boston combined with Rome: (*H*[2] = 10.16, *p* = 0.006)). *Post hoc* tests also revealed a significantly higher improvement in the real/real group as compared to the sham/sham group [Boston: *U* = 58, *z* = 3.04, *p* = 0.001, *r* = 0.76; Boston combined with Rome: (*U* = 115, *z* = 3.13, *p* = 0.001, *r* = 0.64)]. However, while the real/real group was also significantly different form the real/sham group in Boston (*U* = 43, *z* = 2.21, *p* = 0.028, *r* = 0.57), this difference became non-significant after combining the results from Boston with Rome (*U* = 110, *z* = 1.56, *p* = 0.121, *r* = 0.31).

## References

[B1] Ahmed M. A. Darwish E. S. Khedr E. M. El Serogy Y. M. Ali A. M. (2012). Effects of low versus high frequencies of repetitive transcranial magnetic stimulation on cognitive function and cortical excitability in Alzheimer’s dementia. *J. Neurol.* 259 83–92. 10.1007/s00415-011-6128-4 21671144

[B2] Alzheimer’s Association (2012). 2012 Alzheimer’s disease facts and figures. *Alzheimers Dement.* 8 131–168. 10.1016/j.jalz.2012.02.001 22404854

[B3] Ballard C. Gauthier S. Corbett A. Brayne C. Aarsland D. Jones E. (2011). Alzheimer’s disease. *Lancet* 377 1019–1031. 10.1016/S0140-6736(10)61349-9 21371747

[B4] Beekly D. L. Ramos E. M. Lee W. W. Deitrich W. D. Jacka M. E. Wu J. (2007). The National Alzheimer’s coordinating center (NACC) database: the uniform data set. *Alzheimer Dis. Assoc. Disord.* 21 249–258. 10.1097/WAD.0b013e318142774e 17804958

[B5] Belleville S. Clément F. Mellah S. Gilbert B. Fontaine F. Gauthier S. (2011). Training-related brain plasticity in subjects at risk of developing Alzheimer’s disease. *Brain* 134 1623–1634. 10.1093/brain/awr037 21427462

[B6] Bentwich J. Dobronevsky E. Aichenbaum S. Shorer R. Peretz R. Khaigrekht M. (2011). Beneficial effect of repetitive transcranial magnetic stimulation combined with cognitive training for the treatment of Alzheimer’s disease: a proof of concept study. *J. Neural. Transm.* 118 463–471. 10.1007/s00702-010-0578-1 21246222

[B7] Brem A.-K. Atkinson N. J. Seligson E. E. Pascual-Leone A. (2013). Differential pharmacological effects on brain reactivity and plasticity in Alzheimer’s disease. *Front. Psychiatry* 4:124. 10.3389/fpsyt.2013.00124 24109459 PMC3791426

[B8] Brem A.-K. Sensi S. L. (2018). Towards combinatorial approaches for preserving cognitive fitness in aging. *Trends Neurosci.* 41 885–897. 10.1016/j.tins.2018.09.009 30343822

[B9] Buss S. S. Fried P. J. Pascual-Leone A. (2019). Therapeutic noninvasive brain stimulation in Alzheimer’s disease and related dementias. *Curr. Opin. Neurol.* 32 292–304. 10.1097/WCO.0000000000000669 30720478 PMC7659470

[B10] Chang W. H. Kim Y.-H. Yoo W.-K. Goo K.-H. Park C.-H. Kim S. T. (2012). rTMS with motor training modulates cortico-basal ganglia-thalamocortical circuits in stroke patients. *Restor. Neurol. Neurosci.* 30 179–189. 10.3233/RNN-2012-110162 22555430 PMC3589123

[B11] Clare L. Woods R. T. Moniz Cook E. D. Orrell M. Spector A. (2003). Cognitive rehabilitation and cognitive training for early-stage Alzheimer’s disease and vascular dementia. *Cochrane Database Syst. Rev.* 2003:CD003260. 10.1002/14651858.CD003260 14583963

[B12] Cooke S. F. Bliss T. V. P. (2006). Plasticity in the human central nervous system. *Brain* 129 1659–1673. 10.1093/brain/awl082 16672292

[B13] Cotelli M. Calabria M. Manenti R. Rosini S. Zanetti O. Cappa S. F. (2011). Improved language performance in Alzheimer disease following brain stimulation. *J. Neurol. Neurosurg. Psychiatry* 82 794–797. 10.1136/jnnp.2009.197848 20574108

[B14] Di Lorenzo F. Ponzo V. Bonnì S. Motta C. Negrão Serra P. C. Bozzali M. (2016). LTP-like cortical plasticity is disrupted in Alzheimer’s disease patients independently from age of onset. *Ann. Neurol.* 80 202–210. 10.1002/ana.24695 27255833

[B15] Farzan F. Barr M. S. Levinson A. J. Chen R. Wong W. Fitzgerald P. B. (2010). Reliability of long-interval cortical inhibition in healthy human subjects: a TMS-EEG study. *J. Neurophysiol.* 104 1339–1346. 10.1152/jn.00279.2010 20573972

[B16] Ferreri F. Pauri F. Pasqualetti P. Fini R. Dal Forno G. Rossini P. M. (2003). Motor cortex excitability in Alzheimer’s disease: a transcranial magnetic stimulation study. *Ann. Neurol.* 53 102–108. 10.1002/ana.10416 12509853

[B17] Freitas C. Mondragón-Llorca H. Pascual-Leone A. (2011). Noninvasive brain stimulation in Alzheimer’s disease: systematic review and perspectives for the future. *Exp. Gerontol.* 46 611–627. 10.1016/j.exger.2011.04.001 21511025 PMC3589803

[B18] Haffen E. Chopard G. Pretalli J.-B. Magnin E. Nicolier M. Monnin J. (2012). A case report of daily left prefrontal repetitive transcranial magnetic stimulation (rTMS) as an adjunctive treatment for Alzheimer disease. *Brain Stimul.* 5 264–266. 10.1016/j.brs.2011.03.003 22037125

[B19] Hill N. T. M. Mowszowski L. Naismith S. L. Chadwick V. L. Valenzuela M. Lampit A. (2017). Computerized cognitive training in older adults with mild cognitive impairment or dementia: a systematic review and meta-analysis. *Am. J. Psychiatry* 174 329–340. 10.1176/appi.ajp.2016.16030360 27838936

[B20] Huang Y.-Z. Chen R.-S. Rothwell J. C. Wen H.-Y. (2007). The after-effect of human theta burst stimulation is NMDA receptor dependent. *Clin. Neurophysiol.* 118 1028–1032. 10.1016/j.clinph.2007.01.021 17368094

[B21] Huang Y.-Z. Edwards M. J. Rounis E. Bhatia K. P. Rothwell J. C. (2005). Theta burst stimulation of the human motor cortex. *Neuron* 45 201–206. 10.1016/j.neuron.2004.12.033 15664172

[B22] Jacobs H. I. L. Van Boxtel M. P. J. Uylings H. B. M. Gronenschild E. H. B. M. Verhey F. R. Jolles J. (2011). Atrophy of the parietal lobe in preclinical dementia. *Brain Cogn.* 75 154–163. 10.1016/j.bandc.2010.11.003 21130554

[B23] Kakuda W. Abo M. Watanabe S. Momosaki R. Hashimoto G. Nakayama Y. (2013). High-frequency rTMS applied over bilateral leg motor areas combined with mobility training for gait disturbance after stroke: a preliminary study. *Brain Inj.* 27 1080–1086. 10.3109/02699052.2013.794973 23834634

[B24] Kallio E.-L. Öhman H. Kautiainen H. Hietanen M. Pitkälä K. (2017). Cognitive Training Interventions for patients with Alzheimer’s disease: a systematic review. *J. Alzheimers Dis.* 56 1349–1372. 10.3233/JAD-160810 28222505

[B25] Khedr E. M. Abo El-Fetoh N. Ali A. M. El-Hammady D. H. Khalifa H. Atta H. (2014). Dual-hemisphere repetitive transcranial magnetic stimulation for rehabilitation of poststroke aphasia: a randomized, double-blind clinical trial. *Neurorehab. Neural Re.* 28 740–750. 10.1177/1545968314521009 24503205

[B26] Kim C. Choi H. E. Jung H. Lee B.-J. Lee K. H. Lim Y.-J. (2014). Comparison of the Effects of 1 Hz and 20 Hz rTMS on Motor Recovery in Subacute Stroke Patients. *Ann. Rehabil. Med.* 38 585–591. 10.5535/arm.2014.38.5.585 25379487 PMC4221386

[B27] Koch G. Di Lorenzo F. Bonnì S. Ponzo V. Caltagirone C. Martorana A. (2012). Impaired LTP- but not LTD-like cortical plasticity in Alzheimer’s disease patients. *J. Alzheimers Dis.* 31 593–599. 10.3233/JAD-2012-120532 22647254

[B28] Lee J. Choi B. H. Oh E. Sohn E. H. Lee A. Y. (2015). Treatment of Alzheimer’s disease with repetitive transcranial magnetic stimulation combined with cognitive training: a prospective, randomized, double-blind, placebo-controlled study. *J. Clin. Neurol.* 12 57–64. 26365021 10.3988/jcn.2016.12.1.57 PMC4712287

[B29] Liepert J. Bär K. J. Meske U. Weiller C. (2001). Motor cortex disinhibition in Alzheimer’s disease. *Clin. Neurophysiol.* 112 1436–1441. 10.1016/s1388-2457(01)00554-5 11459683

[B30] Mattson M. P. (2004). Pathways towards and away from Alzheimer’s disease. *Nature* 430 631–639. 10.1038/nature02621 15295589 PMC3091392

[B31] McGirr A. Van den Eynde F. Tovar-Perdomo S. Fleck M. P. A. Berlim M. T. (2015). Effectiveness and acceptability of accelerated repetitive transcranial magnetic stimulation (rTMS) for treatment-resistant major depressive disorder: an open label trial. *J. Affect. Disord.* 173 216–220. 10.1016/j.jad.2014.10.068 25462419

[B32] McKhann G. Drachman D. Folstein M. Katzman R. Price D. Stadlan E. M. (1984). Clinical diagnosis of Alzheimer’s disease: report of the NINCDS-ADRDA work group under the auspices of department of health and human services task force on Alzheimer’s disease. *Neurology* 34 939–944. 10.1212/wnl.34.7.939 6610841

[B33] Mori F. Rossi S. Sancesario G. Codecà C. Mataluni G. Monteleone F. (2011). Cognitive and cortical plasticity deficits correlate with altered amyloid-β CSF levels in multiple sclerosis. *Neuropsychopharmacology* 36 559–568. 10.1038/npp.2010.187 20944553 PMC3055691

[B34] Oliveira-Maia A. J. Press D. Pascual-Leone A. (2017). Modulation of motor cortex excitability predicts antidepressant response to prefrontal cortex repetitive transcranial magnetic stimulation. *Brain Stimul.* 10 787–794. 10.1016/j.brs.2017.03.013 28438543 PMC5576557

[B35] Pallanti S. Di Rollo A. Antonini S. Cauli G. Hollander E. Quercioli L. (2012). Low-frequency rTMS over right dorsolateral prefrontal cortex in the treatment of resistant depression: cognitive improvement is independent from clinical response, resting motor threshold is related to clinical response. *Neuropsychobiology* 65 227–235. 10.1159/000336999 22653158

[B36] Pascual-Leone A. Valls-Solé J. Wassermann E. M. Hallett M. (1994). Responses to rapid-rate transcranial magnetic stimulation of the human motor cortex. *Brain* 117(Pt 4), 847–858. 10.1093/brain/117.4.847 7922470

[B37] Preacher K. J. Hayes A. F. (2008). Asymptotic and resampling strategies for assessing and comparing indirect effects in multiple mediator models. *Behav. Res. Methods* 40 879–891. 10.3758/brm.40.3.879 18697684

[B38] Price C. J. Winterburn D. Giraud A. L. Moore C. J. Noppeney U. (2003). Cortical localisation of the visual and auditory word form areas: a reconsideration of the evidence. *Brain Lang.* 86 272–286. 10.1016/s0093-934x(02)00544-8 12921768

[B39] Quan W. X. Zhu X. L. Qiao H. Zhang W. F. Tan S. P. Zhou D. F. (2015). The effects of high-frequency repetitive transcranial magnetic stimulation (rTMS) on negative symptoms of schizophrenia and the follow-up study. *Neurosci. Lett.* 584 197–201. 10.1016/j.neulet.2014.10.029 25449864

[B40] Rabey J. M. Dobronevsky E. (2016). Repetitive transcranial magnetic stimulation (rTMS) combined with cognitive training is a safe and effective modality for the treatment of Alzheimer’s disease: clinical experience. *J. Neural Transm.* 123 1449–1455. 10.1007/s00702-016-1606-6 27631152

[B41] Rabey J. M. Dobronevsky E. Aichenbaum S. Gonen O. Marton R. G. Khaigrekht M. (2013). Repetitive transcranial magnetic stimulation combined with cognitive training is a safe and effective modality for the treatment of Alzheimer’s disease: a randomized, double-blind study. *J. Neural. Transm.* 120 813–819. 10.1007/s00702-012-0902-z 23076723

[B42] Rogalsky C. Matchin W. Hickok G. (2008). Broca’s area, sentence comprehension, and working memory: an fMRI study. *Front. Hum. Neurosci.* 2:14. 10.3389/neuro.09.014.2008 18958214 PMC2572210

[B43] Rossi S. Hallett M. Rossini P. M. Pascual-Leone A. (2009). Safety, ethical considerations, and application guidelines for the use of transcranial magnetic stimulation in clinical practice and research. *Clin. Neurophysiol.* 120 2008–2039. 10.1016/j.clinph.2009.08.016 19833552 PMC3260536

[B44] Rossini P. M. Barker A. T. Berardelli A. Caramia M. D. Caruso G. Cracco R. Q. (1994). Non-invasive electrical and magnetic stimulation of the brain, spinal cord and roots: basic principles and procedures for routine clinical application. Report of an IFCN committee. *Electroencephalogr. Clin. Neurophysiol.* 91 79–92. 10.1016/0013-4694(94)90029-9 7519144

[B45] Rossini P. M. Burke D. Chen R. Cohen L. G. Daskalakis Z. Di Iorio R. (2015). Non-invasive electrical and magnetic stimulation of the brain, spinal cord, roots and peripheral nerves: Basic principles and procedures for routine clinical and research application. An updated report from an I.F.C.N. Committee. *Clin. Neurophysiol.* 126 1071–1107. 10.1016/j.clinph.2015.02.001 25797650 PMC6350257

[B46] Rusjan P. M. Barr M. S. Farzan F. Arenovich T. Maller J. J. Fitzgerald P. B. (2010). Optimal transcranial magnetic stimulation coil placement for targeting the dorsolateral prefrontal cortex using novel magnetic resonance image-guided neuronavigation. *Hum. Brain Mapp.* 31 1643–1652. 10.1002/hbm.20964 20162598 PMC6871247

[B47] Ryu S.-H. Katona C. Rive B. Livingston G. (2005). Persistence of and changes in neuropsychiatric symptoms in Alzheimer disease over 6 months: the LASER-AD study. *Am. J. Geriatr. Psychiatry* 13 976–983. 10.1176/appi.ajgp.13.11.976 16286441

[B48] Sajonz B. Kahnt T. Margulies D. S. Park S. Q. Wittmann A. Stoy M. (2010). Delineating self-referential processing from episodic memory retrieval: common and dissociable networks. *Neuroimage* 50 1606–1617. 10.1016/j.neuroimage.2010.01.087 20123026

[B49] Schrag A. Schott J. M. Alzheimer’s Disease Neuroimaging Initiative (2012). What is the clinically relevant change on the ADAS-Cog? *J. Neurol. Neurosurg. Psychiatr.* 83 171–173. 10.1136/jnnp-2011-300881 22019547

[B50] Silvanto J. Pascual-Leone A. (2008). State-dependency of transcranial magnetic stimulation. *Brain Topogr.* 21 1–10. 10.1007/s10548-008-0067-0 18791818 PMC3049188

[B51] Udupa K. Ni Z. Gunraj C. Chen R. (2009). Interactions between short latency afferent inhibition and long interval intracortical inhibition. *Exp. Brain Res.* 199 177–183. 10.1007/s00221-009-1997-9 19730839

[B52] Wagner T. Eden U. Fregni F. Valero-Cabre A. Ramos-Estebanez C. Pronio-Stelluto V. (2008). Transcranial magnetic stimulation and brain atrophy: a computer-based human brain model study. *Exp. Brain Res.* 186 539–550. 10.1007/s00221-007-1258-8 18193208 PMC3374637

[B53] Wang M. Wong A. H. Liu F. (2012). Interactions between NMDA and dopamine receptors: a potential therapeutic target. *Brain Res.* 1476 154–163. 10.1016/j.brainres.2012.03.029 22472597

[B54] Wang X. Mao Z. Ling Z. Yu X. (2019). Repetitive transcranial magnetic stimulation for cognitive impairment in Alzheimer’s disease: a meta-analysis of randomized controlled trials. *J. Neurol.* 267 791–801. 10.1007/s00415-019-09644-y 31760522

[B55] Wobrock T. Guse B. Cordes J. Wölwer W. Winterer G. Gaebel W. (2015). Left prefrontal high-frequency repetitive transcranial magnetic stimulation for the treatment of schizophrenia with predominant negative symptoms: a sham-controlled, randomized multicenter trial. *Biol. Psychiatry* 77 979–988. 10.1016/j.biopsych.2014.10.009 25582269

[B56] Yang Y.-R. Tseng C.-Y. Chiou S.-Y. Liao K.-K. Cheng S.-J. Lai K.-L. (2013). Combination of rTMS and treadmill training modulates corticomotor inhibition and improves walking in Parkinson disease: a randomized trial. *Neurorehabil. Neural. Repair.* 27 79–86. 10.1177/1545968312451915 22785003

