# Supplementary material for: Corticomotor Plasticity Predicts Clinical Efficacy of Combined Neuromodulation and Cognitive Training in Alzheimer’s Disease
Source: Front Aging Neurosci. 2020 Jul 8;12:200. doi: 10.3389/fnagi.2020.00200 (PMC7360860; doi:10.3389/fnagi.2020.00200)
Supplement: TABLE S1 — Plasticity measures in the AD sample from Boston (n = 21) at baseline and after the end of the intervention (T5, T10, T20, T30). [file Presentation_1.pdf]

Supplemental Table S1. Plasticity measures in the AD sample from Boston (n=21) at baseline and after the end of the intervention (T5, T10, T20, T30).

		T5 pre	T10 pre	T20 pre	T30 pre	T5 post	T10 post	T20 post	T30 post
**Real/ real**	Mean	0.94	0.97	1.19	1.06	1.08	1.19	1.17	1.10
	SD	0.36	0.54	1.46	0.56	0.60	0.43	0.48	0.41
**Sham/ sham**	Mean	0.86	0.99	1.12	1.00	1.38	1.28	1.50	1.16
	SD	0.19	0.26	0.37	0.26	0.29	0.38	0.70	0.40
**Real/ sham**	Mean	1.49	1.37	1.17	1.26	1.48	1.17	1.54	1.30
	SD	0.41	0.56	0.52	0.50	0.94	0.68	0.89	0.82

Supplemental Table S2. ADAS-Cog results from each site (Boston and Rome) and combined results.

		Real/rea		Sham/sham		Real/sham	
		Mean	SD	Mean	SD	Mean	SD
**Boston**	Pre	22.60	10.90	22.81	12.48	25.20	10.46
	Post	20.77	11.95	23.48	10.08	25.39	10.22
	FU	17.80	9.11	-	-	24.34	8.97
	N	10	10	6	6	5	5
**Rome**	Pre	23.67	8.98	26.00	9.90	25.00	10.05
	Post	20.83	11.07	26.00	9.90	24.00	11.34
	FU	21.33	9.14	23.50	6.363961	22.40	13.90
	N	6	6	2	2	5	5
**Total**	Pre	23.00	2.48	23.61	3.99	25.10	3.06
	Post	20.79	2.81	24.11	3.31	24.70	3.23
	FU	19.13	2.25	23.50	4.50	23.37	3.50
	N	16	16	8	8	10	10

Supplemental results

Before adding the results from Rome to the results from Boston, we find very similar results. In Boston, the largest score change in ADAS-Cog within one month after the end of intervention was significantly different between groups (Boston: $H[2]=11.626$, $p= 0.003$; Boston combined with Rome: ($H[2]=10.16$, $p=0.006$)). Post-hoc tests also revealed a significantly higher improvement in the real/real group as compared to the sham/sham group (Boston: $U = 58$, $z = 3.04$, $p = 0.001$, $r = 0.76$; Boston combined with Rome: ($U=115$, $z=3.13$, $p=0.001$, $r=0.64$)). However, while the real/real group was also significantly different form the real/sham group in Boston ($U = 43$, $z = 2.21$, $p = 0.028$, $r = 0.57$), this difference became non-significant after combining the results from Boston with Rome ($U=110$, $z=1.56$, $p=0.121$, $r=0.31$).
